# Development of Auditory Selective Attention: Why Children Struggle to Hear in Noisy Environments

**DOI:** 10.1037/a0038570

**Published:** 2015-03

**Authors:** Pete R. Jones, David R. Moore, Sygal Amitay

**Affiliations:** 1 MRC Institute of Hearing Research, Nottingham, United Kingdom, and UCL Institute of Ophthalmology; 2 MRC Institute of Hearing Research, and Cincinnati Children’s Hospital Medical Center, Cincinnati, Ohio; 3 MRC Institute of Hearing Research

**Keywords:** auditory masking, selective attention, internal noise, pure tone detection, reverse correlation

## Abstract

Children’s hearing deteriorates markedly in the presence of unpredictable noise. To explore why, 187 school-age children (4–11 years) and 15 adults performed a tone-in-noise detection task, in which the masking noise varied randomly between every presentation. Selective attention was evaluated by measuring the degree to which listeners were influenced by (i.e., gave weight to) each spectral region of the stimulus. Psychometric fits were also used to estimate levels of internal noise and bias. Levels of masking were found to decrease with age, becoming adult-like by 9–11 years. This change was explained by improvements in selective attention alone, with older listeners better able to ignore noise similar in frequency to the target. Consistent with this, age-related differences in masking were abolished when the noise was made more distant in frequency to the target. This work offers novel evidence that improvements in selective attention are critical for the normal development of auditory judgments.

Whether a teacher’s voice or an oncoming car, the ability to detect an auditory signal in a noisy environment is vital for everyday life. When the background noise is repetitive and predictable, children are often as good as adults at detecting an auditory signal (Oh, Wightman, & Lutfi, 2001; Wightman, Callahan, Lutfi, Kistler, & Oh, 2003). However, when the background noise is chaotic and unpredictable, children’s hearing declines markedly. For example, in the presence of a noise that varies randomly between every presentation, 4- to 5-year-old children require a fivefold increase in signal intensity relative to adults (Oh et al., 2001). This puts children at risk of missing crucial information.

The masking caused by an unpredictable stimulus is particularly interesting for cognitive scientists because it cannot be explained purely by peripheral auditory mechanisms (see Kidd, Mason, Richards, Gallun, & Durlach, 2007; Leibold, 2012, for reviews). Thus, it occurs even when signal and masker are both clearly audible (Brungart, 2001), and—unlike simple, *energetic* masking (Fletcher, 1940)—children’s difficulties persist even when the background noise is separated from the target in time (Hall, Buss, & Grose, 2005; Leibold & Neff, 2007), space (Hall et al., 2005; Wightman et al., 2003), or spectral content (Oh et al., 2001). Moreover, compared with masking by more predictable stimuli, the effects are often orders of magnitude greater. For example, consider the case (shown graphically in Figure 1) where a listener is trying to detect a fixed-frequency pure tone, masked by four other “distractor” tones of variable frequencies (e.g., Leibold, Hitchens, Buss, & Neff, 2010). If the frequency of each distractor tone is held constant within each test block, then amounts of masking are relatively small. However, if the distractor frequencies vary on every presentation (e.g., from trial-to-trial, and from interval-to-interval within each trial), then amounts of masking can be 20–30 dB greater (3 dB corresponding to a doubling of signal power). Notably, this difference in masking holds even if the same overall set of distractor stimuli are used in both cases (see Figure 1). Why then is it that children struggle so profoundly to detect auditory signals when the background environment is unpredictable?[Fig-anchor fig1]

Two potential mechanisms are often hypothesized to underlie sensory development. One possibility is that children suffer from greater internal noise. Internal noise is random error in the decision process, arising from sources intrinsic to the listener (Macmillan & Creelman, 2005). It may be caused, for example, by stochastic neural activity (Javel & Viemeister, 2000), blood flowing near the inner ear (Soderquist & Lindsey, 1971), and random fluctuations in memory or motivation.^1^ The presence of internal noise causes responses to become inconsistent (Green, 1964) and performance to diminish (Jones, Shub, Moore, & Amitay, 2013).

Alternatively, it may be that children experience greater masking because they attend less selectively to the task-relevant information. Thus, most perceptual tasks involve multiple sources of information, distributed, for example, across different features of the stimulus (Hillis, Watt, Landy, & Banks, 2004), between sensory modalities (Ernst & Banks, 2002), or across multiple observations of the same stimulus over time (Swets, 1959). In an auditory masking task, each spectral region can be thought of as a potential information channel, and the listener ought to attend only to those spectral regions liable to contain the signal. For example, in the present study, the task was to listen for a 1-kHz tone. Accordingly, observers should have attended to information distributed around 1-kHz, and ignored any energy (i.e., distractor tones) higher or lower in frequency. Some observers may fail to do this, and may instead pay attention (i.e., “give weight”) to spectral regions containing only noise. Such observers would be prone to be misled by irrelevant variations in the stimulus, and are said to have a less efficient decision strategy. It may be that children are particularly poor in this regard, and that their increased masking therefore reflects an inappropriate weighting of task-relevant/-irrelevant information. Note that if the overweighted regions lay adjacent to the signal, then this would be equivalent to children having a broader “attention band” (or “attention spotlight”; Posner & Petersen, 1990).

## Previous Literature on Internal Noise and Selective Attention

In the general auditory literature, internal noise and selective attention have each been invoked to explain various aspects of sensory development. For example, Buss, Hall, and Grose (2009) observed that children were poorer than adults at discriminating differences in tone intensity. The authors attributed this to greater levels of internal noise, and cited as evidence the shallower psychometric functions exhibited by the children. In a similar vein, Allen and Nelles (1996) conducted a sample discrimination task, in which the listener was asked to categorize tonal sequences, the frequencies of which were drawn from one of two overlapping distributions. Children were poorer at “integrating information” (e.g., showed less improvement as sequence length increased) and, by fitting a model to performance as a function of sequence length, it was shown that this could be explained by a greater magnitude of internal noise in children. Children’s poorer thresholds on tone-in-noise detection (Buss, Hall, & Grose, 2006), backward masking (Hill, Hartley, Glasberg, Moore, & Moore, 2004), and speech-in-noise comprehension (Stuart, 2008) tasks have been attributed similarly to elevated levels of internal noise.

Other authors have stressed the importance of selective attention during auditory development. For example, in the probe−signal paradigm, listeners attempt to detect a pure tone. On most trials, the frequency of the tone is constant (the “standard”). However, on a small proportion of trials, a “probe” tone is presented at a different frequency (Greenberg & Larkin, 1968). To the extent that listeners fail to detect these unexpected probes, they can be said to be listening selectively to the spectral region containing the standard. Crucially, children around seven years old have been shown to perform similarly to adults (Greenberg, Bray, & Beasley, 1970), whereas 7- to 9-month-old infants exhibit broader tuning curves (Bargones & Werner, 1994)—responding to off-frequency probes almost as reliably as to the standard. This suggests that hearing does indeed become more selective during development, and that this change happens between the first and seventh year of life. Such a conclusion is consistent with other findings within the auditory literature; for example, younger children show poorer behavioral (Wightman & Kistler, 2005) and electrophysiological (Coch, Sanders, & Neville, 2005) responses to speech when competing words are presented in the contralateral ear (see Leibold, 2012, for an overview). Furthermore, that children’s deficits are primarily attentional is also consistent with an extensive general literature on selective attention, which indicates that, for example, young children are poorer at recalling information from among competing talkers (Määttä, Pääkkönen, Saavalainen, & Partanen, 2005), at switching between tasks predicated on different stimulus properties (Brace, Morton, & Munakata, 2006), at learning the relevant features in an unsupervised learning task (Brooks, Hanauer, Padowska, & Rosman, 2003), or at quickly sorting items that vary across both task-relevant and task-irrelevant dimensions (see Hanania & Smith, 2010; Ruff & Rothbart, 1996, for reviews).

Within the specific literature concerning hearing-in-noise, the tendency has been to attribute children’s difficulties solely to deficits in selective attention (e.g., Leibold et al., 2010; Lutfi, Kistler, Oh, Wightman, & Callahan, 2003; Oh et al., 2001; Wightman, Kistler, & O’Bryan, 2010). The evidence to support this has come primarily from a mathematical model known as component relative entropy (CoRE; Lutfi, 1993; Oh & Lutfi, 1998). In this model, any masking that cannot be accounted for by purely peripheral mechanisms (i.e., energetic masking) is explained by either the number and/or the range of information channels to which the observer attends. Based on fits of behavioral data to this model, children’s greater masking has been attributed to an increase in one (Oh et al., 2001) or both (Lutfi et al., 2003) of these factors. Though highly suggestive, such fits are not, however, a strong test of the underlying mechanisms that limit performance, because selective attention is effectively the only free parameter in the CoRE model. Thus, observed changes in performance could equally be explained by a different model in which internal noise was allowed to vary between children and adults. And, by the same logic, the earlier results of Allen and Nelles (1996) could equally be explained by a model in which internal noise was constant and children differed in how they attended to the various items in the sequence. What is lacking, therefore, is either a behavioral model in which both internal noise and selective attention are represented independently, or an experimental paradigm in which the predictions of these two hypotheses differ (Experiments 2 and 3 of the present article, respectively).

Recently, within the visual literature, concerted attempts *have* been made to delineate the effects of internal noise and selective attention. This has been done during perceptual learning (Lu & Dosher, 2008, 2009), development (Manning, Dakin, Tibber, & Pellicano, 2014), and aging (Bogfjellmo, Bex, & Falkenberg, 2013). Generally, these studies have tended to report changes in both internal noise and selective attention (though cf. Gold, Bennett, & Sekuler, 1999). For example, Manning et al. (2014) examined why children are less able to judge the mean direction of a cloud of coherently moving dots. A technique known as equivalent noise analysis was used, in which a known level of external noise was manipulated, in order to “titrate” unknown levels of internal noise and decision efficiency (see Dakin, Mareschal, & Bex, 2005; Hurlbert, 2000). Based on this, it was shown that children were limited by both internal noise and their ability to combine information across information channels. Notably, and in contrast to the present work, the method used measured only the efficiency of the observer’s decision strategy, not the strategy itself (i.e., not what information children were using to make their decisions). Relatively few predictions could therefore be made on what changes to the stimulus would exacerbate or ameliorate children’s difficulties. Conversely, in the present study, we attempted to use the technique of reverse correlation to measure exactly what information observers based their sensory judgments.

To sum up, previous evidence has provided no strong prediction as to whether children’s difficulties hearing-in-noise are due to changes in internal noise and/or selective attention. The general developmental literature contains strong claims of each, but the two have seldom been compared directly within a single study. The specific literature on auditory masking has favored selective attention in its accounts of development, but again no direct comparisons have been made, either experimentally or in terms of models fitted to the data. In the visual psychophysical literature, direct comparisons have been made between internal noise and selective attention. The results there seem to indicate that changes in both may be important during development. However, the results are not conclusive, and it is not clear whether these results generalize to audition. Furthermore, even within the existing visual literature, it is unclear precisely how it is that children’s decisions improve with age.

## The Present Study

The present study aimed to evaluate whether changes in internal noise and/or selective attention can explain the development of hearing in unpredictable noise. It further aimed to quantify how listening strategies differ between younger and older children.

Distinguishing between internal noise and selective attention is made problematic by the fact that classic metrics, such as the slope of the psychometric function, confound both sources of inefficiency. Thus, psychometric slopes are flattened either if the listener attends to uninformative aspects of the stimulus, or if internal noise magnitude increases. To separate these two processes, in the present study, we used a two-step approach, outlined previously by Berg (2004).

In the first step, a trial-by-trial analysis technique known as reverse correlation was used to measure spectral weights (see Dai & Micheyl, 2010). To understand reverse correlation, consider that an unpredictable noise will, by definition, differ on every trial. The information contained within each part of the stimulus (in this case, the relative amount of acoustic energy contained within each spectral region) will therefore vary trial-by-trial. By comparing (e.g., via correlation or regression) these trial-by-trial stimulus variations with the observer’s trial-by-trial responses, the relative level of importance (“weight”) given to each region can be determined (see Figure 2). For example, in the present study, a large positive weight would indicate that the listener consistently responded in favor of the interval with more energy in the corresponding spectral region, whereas a large negative weight would indicate the inverse: that the listener tended to select the interval with *less* energy at that frequency. In this way, “[the] weight associated with a particular component can be used to measure the extent to which the observer attends to that component” (Dai & Berg, 1992, p. 1354). The overall pattern of weights provides a measure of the observer’s decision strategy, which in turn can be compared with the ideal in order to compute a measure of *decision efficiency* (see Figure 2). In general terms, the noteworthy aspect of this approach is that, whereas gross (“molar”) metrics such as *d*′, threshold, and percent-correct provide a measure of how *well* an observer is performing, this trial-by-trial (“molecular”) analysis tells us *how* the observer is performing the task. [Fig-anchor fig2]

In the second step, psychometric functions were fitted to the parameters of the physical stimulus, *after* it had been filtered by the listener’s estimated decision strategy (i.e., the spectral weights). By fitting to the weighted input, rather than the raw physical values, the relative efficiency of the weighting strategy was partialed out of the psychometric fit, and the slope parameter could be interpreted as an unambiguous index of internal noise magnitude. (For further details, see the Experiment 2 Method section.)

Three experiments were carried out. Experiment 1 investigated the developmental trajectory of hearing in unpredictable noise, and asked by what age performance is generally adult-like. This experiment was similar to earlier experiments by Oh, Wightman, and colleagues (e.g., Oh et al., 2001). However, it was necessary to establish that differences in masking could be reliably elicited using relative few trials and the heterogeneous (e.g., age, socioeconomic status [SES]) cohort of children recruited to the present study. It was also needed to inform the choice of stimuli and age groups used in the subsequent experiments. Experiment 2 investigated whether changes in selective attention or internal noise could explain the maturation of masking, using the two-step modeling approach just described above. The results indicated that changes in selective attention alone could explain the maturation. Experiment 3 tested a key corollary of this result, by examining whether masking decreased as the spectral proximity between target and noise was decreased. This showed that excluding noise from around the target signal selectively attenuated masking in younger listeners, confirming the conclusion from Experiment 2 that young children are less able to listen selectively. Finally, a multiple regression was conducted, using the combined data from Experiments 2 and 3, to examine what factors predict individual differences in masking. This indicated that variations in SES may in particular affect children’s ability to hear in noise.

## General Methods

Here we describe those methods that were common across all experiments.

### Listeners

The total cohort consisted of 187 children aged 4–11 years old and 15 adults with normal hearing (see Table 1 for details). Each listener only participated in one experiment, and data gathering took place across 3 years (2010–2012).[Table-anchor tbl1]

Children were recruited through Nottingham University’s Summer Scientist week: a public engagement event in which local children visit the university to participate in a range of scientific studies (http://www.summerscientist.org/). Due to time constraints, children were not screened for hearing difficulties in advance, but data from 23 listeners were excluded post hoc, based on their 1-kHz pure tone thresholds (>20 dB hearing level [HL]). Note that single-tone screening at 1 kHz has been demonstrated to provide a relatively robust method of screening for hearing impairments (Maxwell & Davidson, 1961). These thresholds were calculated using the adaptive tracking procedure described in the General Methods: Procedure section, as part of the main experiment. Two of the excluded children exhibited moderate hearing loss (>40 dB HL) and were already receiving clinical care. The remaining excluded children exhibited thresholds consistent with mild hearing loss (<40 dB HL), which may have reflected a mixture of sensory (e.g., otitis media) and nonsensory (e.g., fatigue, general noncompliance) factors (see Appendix A in the Supplemental Materials for further details). Twenty-one additional children were excluded from the analyses in Experiments 1−3 because they did not complete every condition (Experiments 1 and 2) or completed fewer than three blocks (Experiment 3).

In Experiment 1, 15 adult listeners (*M*_age_ = 26.0 years; 3 male) were also recruited through advertisements placed around the Nottingham University campus. These listeners had audiometrically normal hearing (≤20 dB HL bilaterally at 0.25−8 kHz octaves; British Society of Audiology, 2004), and received an inconvenience allowance of £7.5 per hr for their time.

Written consent was obtained from all participants (adults) or the responsible caregiver (children), and children gave verbal assent to participate. The study was conducted in accordance with Nottingham School of Psychology Research Ethics Committee approval.

### Procedure

Listeners performed a cued, two-interval, two-alternative forced choice, fixed-target, tone detection task. On each trial, the target signal was a 1 kHz pure tone, which was randomly assigned with equal probability to one of two temporal intervals. The listener’s task was to indicate in which interval the target signal had occurred. This was couched as a game in which the player must “listen for where the special alien [sound] is hiding.” In signal plus noise trials, an independent noise sample was presented in each interval, and the listener was encouraged to “ignore the aliens that are trying to distract you.”

Each trial began with a 400-ms reminder of the target tone, presented at a constant level of 66 dB sound pressure level (SPL). This cue was similar to that used previously by Wightman et al. (2003), and was intended to encourage listeners to use a consistent listening strategy (specifically, to prevent listeners from forgetting the sound of the target signal as the adaptive track approached threshold). After a 700-ms pause, the two stimuli were presented for 370 ms each, separated by a 500-ms interstimulus interval (see Figure 3). Listeners then had an unlimited amount of time to respond using a button box, before being presented with 1,000 ms of feedback, consisting of a “happy” (correct) or “sad” (incorrect) cartoon face, and a corresponding sound. Throughout the experiment, child-friendly graphics were presented on an LCD screen (see video in supplemental materials).[Fig-anchor fig3]

Within each test block, a two-down one-up adaptive track (Levitt, 1971) was used to measure the observer’s 71% correct detection limen (DL), either in quiet or in the presence of the multitone masker. The level of the target tone was initialized at 66 dB SPL. It was then adapted up or down in 6 dB steps, reducing to 3 dB steps after the second reversal. Each block was terminated after four reversals at 3 dB steps, or after 35 trials (whichever occurred first).

Before testing, listeners were required to answer correctly one practice trial in quiet and three practice trials in noise. In the majority of cases, this was achieved with no errors, but some children required five or six trials to reach this criterion (*M*_*n*trials_ = 4.4). During practice, the task demands were highlighted by: fixing the target level at a high intensity (66 dB SPL), increasing all stimulus durations to 800 ms, and attenuating any noise levels to 45 dB SPL.

### Stimuli and Apparatus

**Figure 1 fig1:**
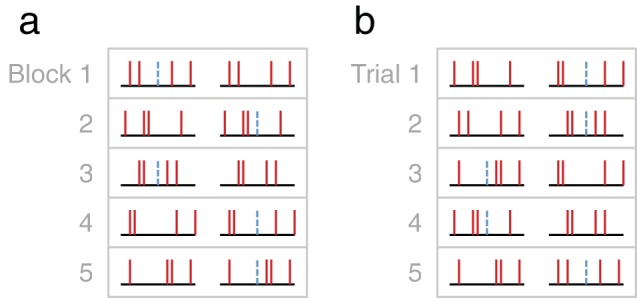
Schematic illustration of “predictable” (a) and “unpredictable” (b) stimulus configurations (given in the same format as the stimuli for the present study, shown in Figure 3a). Each trial consists of two, sequential stimulus observations, with the target stimulus randomly presented in either the first or the second interval. Distractor frequencies are shown in red, and the target frequency is shown in dashed blue. In the predictable case (a), the distractors are constant within each trial/block, and expected masking is low. In contrast, in the unpredictable case (b), the distractors vary randomly on every presentation, and expected masking is high. Note, however, that in both cases (a and b), the overall set of stimuli is identical. See the online article for the color version of this figure.

The stimuli (see Figure 3) were similar to those used in a number of previous studies (e.g., Oh & Lutfi, 1998; Oh et al., 2001). The target signal was always a 1-kHz sinusoid, 370 ms in duration including 20-ms cos^2^ on/off ramps. In noise conditions, a multitone complex was also presented at each interval, comprised of *N* distractor tones. The noise was 370-ms long, including 20-ms cos^2^ ramps, and, in the target interval, it was presented simultaneously with the target tone. The frequency, phase, and relative amplitude of each distractor was independently randomized prior to every presentation (i.e., between intervals, as per Figure 1b). Phases and amplitudes were drawn from rectangular and Rayleigh random distributions, respectively. Frequencies were drawn from a rectangular distribution (linearly distributed in Experiment 1; logarithmically distributed in Experiments 2 and 3), with a protected region centered on the signal frequency, designed to reduce (though not eliminate) energetic masking. The precise width of the protected region varied across experiments, but was always greater than one equivalent rectangular band (Glasberg & Moore, 1990). The level of the target tone varied between 0 and 80 dB SPL, according to an adaptive track (see the Procedure subsection above). The noise was always presented at an overall total level of 60 + ε dB SPL, where ∈ was a random variable, uniformly distributed between −3 and 3. This jitter was applied independently to every noise sample, and was designed to discourage listeners from responding to overall loudness.

Stimuli were digitally synthesized in Matlab, Version 7.4 (The MathWorks, Natick, MA) using a sampling rate of 44.1 kHz and 24-bit quantization. Digital-to-analog conversion was carried out by an external USB sound card (Experiments 1 and 2: custom-built in-house hardware; Experiment 3: M-Audio Fast Track Pro), interfaced using the Psychophysics Toolbox, Version 3 (Brainard, 1997; Pelli, 1997) ASIO wrapper (Steinberg Media Technologies, Hamburg, Germany). The stimuli were presented monaurally to the left ear, using Sennheiser HD 25-I headphones (lightweight, closed-back, supra-aural headphones), which exhibit good linearity and are comfortable for children.

Testing occurred in sound-attenuating booths, with an experimenter present to provide instruction and encouragement. A minority of children were accompanied by a caregiver (generally their parent), who sat outside the child’s field of vision and could not hear the stimuli.

### Measures

The 71% correct DLs were determined by averaging the signal level at the last two reversals of the adaptive track. Masking level was calculated for each noise block, as DL − DL_quiet_, where DL_quiet_ was the listener’s mean DL across signal-only blocks.

With the children (but not the adult controls), three additional, more general measures were taken. Everyday attention was assessed using the Strengths and Weaknesses of ADHD symptoms and Normal behavior (SWAN) Scale (Swanson et al., 2006), an 18-item parental questionnaire concerning a child’s ability to regulate his or her behavior and pay attention. SES was assessed using the 2010 U.K. Government’s Index of Multiple Deprivation, which is linked to the postcode of the child’s residence (McLennan et al., 2011). Vocabulary was assessed using the British Picture Vocabulary Scale (BPVS; Dunn, Dunn, Whetton, & Burley, 1997).

## Experiment 1: Developmental Differences in Masking

The purpose of this experiment was to establish whether a wide cohort of 5- to 11-year-old children could perform the masking task (see Figure 3) with minimal practice, and whether differences in masking could be reliably detected between younger and older children. Such differences have previously been demonstrated between preschool children (< 6 years old) and adults (Oh et al., 2001; Wightman & Allen, 1992), and have been explored within small cohorts of older children by Lutfi et al. (2003) and Leibold and Neff (2007). This experiment also informed the stimuli used in the subsequent two experiments.

### Method

Each masker consisted of *N* distractor tones, randomly selected from a band-passed Gaussian noise (0.1–10 kHz), as per Neff and Callaghan (1988). On each observation interval, one of 50 pregenerated noise samples was randomly selected, and a fast Fourier transform was used to decompose the noise into 2.7-Hz spaced spectral components. Any components falling within a 160-Hz notch arithmetically centered on the target frequency (the protected region) were removed, and *N* of the remaining components was randomly selected to form the multitone complex. Thus, the candidate tones were uniformly spaced between 100 and 10000 Hz on a linear scale, and selecting all components (≈ 3500) was equivalent to synthesizing a notched broadband noise (BBN).

The number of distractor tones (*N*) followed the sequence: 0, 2, 10, 30, 300, 906, and ≈ 3500, with each condition presented in a separate block. The order of the blocks was intended to ensure that task difficulty increased gradually, but may have introduced order effects (see Experiment 1: Results and Discussion).

### Results and Discussion

Children generally appeared to grasp the task rapidly, and all of them completed the practice stage. That the children understood the task was supported by their detection performance in quiet, which did not differ significantly from that of adults (unbalanced one-way analysis of variance [ANOVA]), *F*(52) = 1.09, *p* = .360 (see Appendix A in Supplemental materials for tone-in-quiet thresholds across all experiments). In the noisy conditions, by contrast, children exhibited substantially poorer performance (i.e., more masking) than adults (see Figure 4). This was confirmed by a mixed-effects ANOVA, where there was a significant between-listener effect of age on DL_noise_, *F*(3, 52) = 5.58, *p* = .002, η_p_^2^ = 0.24. Masking also varied within listeners as the number of distractors was varied, *F*(5, 260) = 20.26, *p* < .001, η_p_^2^ = 0.90. Greatest mean masking occurred for all ages when the noise contained 30 distractor components (see Figure 4). The nonmonotonic relation between number of distractors and amount of masking is consistent with previous studies, which have also reported maxima at 20–40 components (e.g., Lutfi et al., 2003; Oh & Lutfi, 2000; Oh et al., 2001).[Fig-anchor fig4]

The overall performance of the oldest children (9–11 years old) was statistically indistinguishable from that of adults, *F*(1, 162) = 1.82, *p* = .179, suggesting that the ability to filter out unpredictable distractors is largely mature by adolescence. A post hoc comparison did indicate a specific difference in the BBN condition, where the 9- to 11-year-old children were about 8 dB poorer than adults, *t*(26) = 2.31, *p* = .029. However, this effect was not subsequently replicated in Experiment 3, and may therefore relate to an observed loss of attention in several children (this condition having always been tested last in the session). The 7- to 8-year-olds exhibited intermediate amounts of masking, but were generally more similar to the older children than to the younger children. All age groups exhibited markedly less masking than the preschool children reported by Oh et al. (2001), indicating substantial development during early childhood.

Differences in performance were also observed between the adult group and the adult data reported by Oh et al. (2001). In particular, masking in the present study varied less with the number of distractors (i.e., the functions in Figure 4 were flatter than those reported by Oh et al., 2001). This may have been due to differences in how thresholds were computed (in the present study, we used the last two reversals of a single adaptive track; Oh et al., 2001, used a psychometric fit to three independent runs). Alternatively, it may have been due to learning effects. Unlike Oh et al. (2001), in Experiment 1, blocks followed a constant order, with maskers becoming progressively denser throughout the session. Because learning is known to occur on this task (Jones, Moore, Shub, & Amitay, 2014, see also Neff & Callaghan, 1988; Neff & Dethlefs, 1995), masking may have been inflated for low *N* distractors, where masking is typically low, and deflated for higher *N* distractors, where masking is typically high. If this was the case, masking would be expected to be greater than shown by Oh et al. (2001) at low *N*, and less at high *N*. The observed data were consistent with this pattern.

In summary, the ability to detect signals in complex and unpredictable environments is essential for everyday life. Using a tone detection task, this ability was shown to improve between 4 and 11 years, by which point performance was adult-like. This finding is consistent with Lutfi et al. (2003), who observed elevated masking at 6–10 years and adult-like levels at 11–16 years, and is also consistent with earlier work by Oh et al. (2001) and Wightman et al. (2003). The time course also parallels that of other basic auditory tasks such as temporal, spectral, and binaural judgments, all of which have been observed to mature by around eight to eleven years (D. R. Moore, Cowan, Riley, Edmondson-Jones, & Ferguson, 2011; Sanes & Woolley, 2011). This suggests that common developmental changes may be taking place throughout the auditory system (seeJ. K. Moore, 2002), or in the wider decision-making networks that the auditory system informs.

## Experiment 2: Weight Profiles and Internal Noise

Experiment 1 demonstrated that younger children exhibit greater masking with unpredictable stimuli. Experiment 2 investigated why this is the case. Estimates of selective attention, internal noise magnitude, and bias were computed, and were compared across age groups. Because Experiment 1 indicated relatively little difference in masking among children more than 7 years old, Experiment 2 compared only “younger” (4–7 years old) and “older” (8–11 years old) children (see Table 1 for details).

### Method

#### Stimuli and design

The stimuli in Experiment 1 were selected for consistency/comparison with previous studies. However, because noise components were distributed linearly in frequency, more energy was expected to lie above the (geometrically centered) target. This meant that listeners could potentially use overall pitch as a cue. Accordingly, in Experiment 2, the distractor frequencies were distributed on a log scale, with a one-third octave notch around the target (891–1120 Hz). To maximize the masking effect, the range of frequencies was also reduced, to 223–4490 Hz.

Because a large number of trials were required to perform the reverse correlation analysis, only a single noise condition (*N* = 30) was used throughout, though a new, random noise sample continued to be generated on every presentation. This number of components represents a balance between maximizing the variability of the stimulus, and preventing the stimuli from becoming too sparse to perform the correlational analysis. It also permitted direct comparison with the data of Jones, Moore, et al. (2014). Note that there is controversy in the auditory literature as to precisely what degree such a noise may be termed an “informational” versus an “energetic” masker (e.g., see Durlach et al., 2003). This has no bearing on the present work, however, and we make no claims either way.

Because relatively few distractor tones were required, noise complexes were constructed by simply summing up 30 pure tones of random frequency, phase, and amplitude. Phase and amplitude were uniformly and Rayleigh randomly distributed (as before), and frequencies were drawn without replacement from a pool of 715 log-spaced candidates.

The first test block was always performed in quiet. Listeners then completed as many masked tracks as they felt comfortably able, up to a maximum of eight (*M*_blocks_ = 5.0, *SD* = 1.6).

#### Analysis

**Figure 2 fig2:**
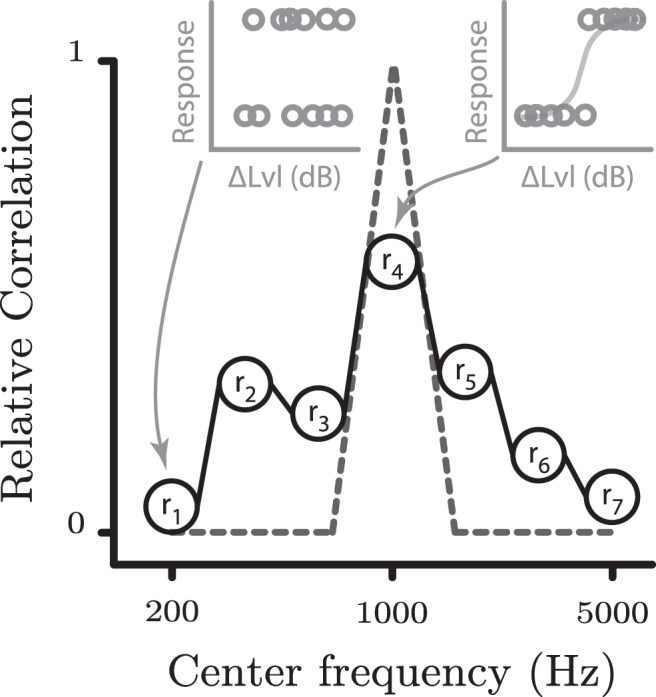
Schematic illustration of weight measurements. The *r* values represent the relative degree to which the observer’s response was determined by each feature. In the present study, this was quantified by computing multiple regression coefficients, and normalizing them so that their magnitudes sum to 1. Efficiency was computed as 1 minus the root mean square difference from the ideal (thin dashed line). For further details, see the Experiment 2 Method section.

**Figure 3 fig3:**
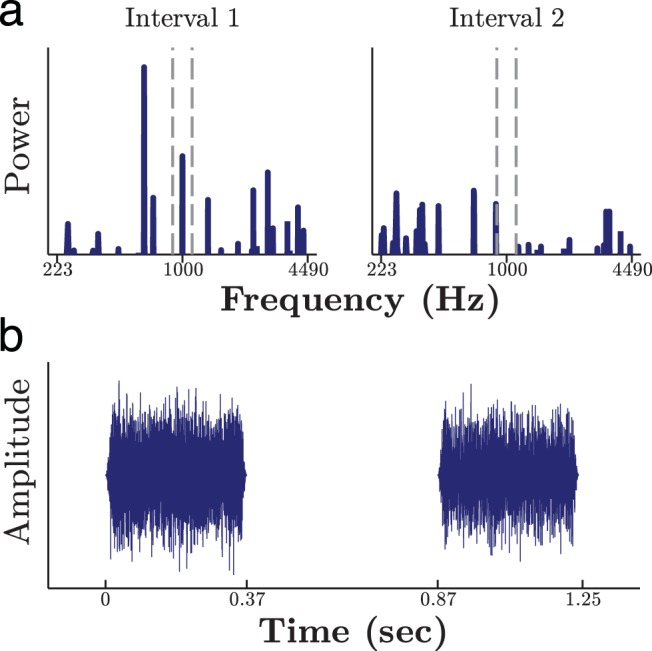
Example stimuli, shown in both the frequency (a) and the time (b) domains. Here, the target tone is in the first interval, and it is masked by a 30 component complex presented simultaneously (the target may not be discernible in the time domain). The number (*N*) of distractors was varied across conditions, and their precise frequencies, phases, and relative amplitudes were randomized prior to every presentation. The dotted vertical lines denote the protected frequency region, within which masker energy was not permitted. Note, in Experiment 1 (only), the noise components were linearly spaced and the spectral range was larger than shown here. See the online article for the color version of this figure.

**Figure 4 fig4:**
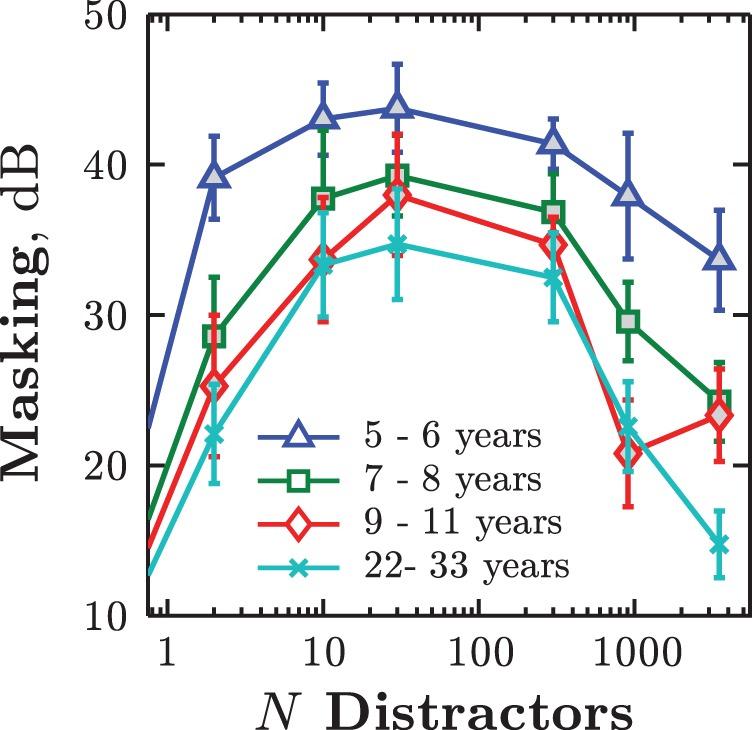
Experiment 1: Group mean masking (± 1 *SE*) as a function of *N* distractors, for each age group (lines). Masking was calculated by subtracting the listener’s detection threshold in quiet from their detection threshold in noise (see the Method section). The curve on the left extends toward the *N* = 0 (quiet) condition, where, by definition, masking equaled 0. At approximately 3,500 distractors, the noise was equivalent to a notched broadband noise. See the online article for the color version of this figure.

Relative weights were computed using the same reverse correlation method used previously by Alexander and Lutfi (2004) and Jones, Moore, et al. (2014). In short, logistic multiple regression (Matlab’s GLMFIT) was used to predict the listener’s responses based on the trial-by-trial variations in the stimulus. The listener’s response was the dependent variable (irrespective of whether it was correct or incorrect). Each independent variable was the difference in level between the two stimulus observations within each trial, as computed for each of seven, uniformly spaced, one-third octave bins. To derive relative weights, the estimated regression coefficients were then normalized so that their magnitudes summed to 1. Ideally, the observer should give weight only to the central, target region. But in practice, an observer may also be influenced by purely noisy components of the stimulus. The overall efficiency of the weight vector was calculated by computing as 1 minus the root mean square difference between the observed values and the ideal (see Figure 2). This yielded one estimate of weight efficiency per listener (where 1 indicated perfect efficiency/selectivity).

This process of deriving weights was suboptimal—though not unprecedented (Alexander & Lutfi, 2004; Jones, Moore, et al., 2014)—because the variability in the target bin (introduced by adapting the target) was not identically distributed to the variability in the noise bins (introduced by Rayleigh jitter). This discrepancy in variance between bins may have introduced some noise into the weight measurements (see Richards & Zhu, 1994). However, Monte Carlo simulations indicated that the additional measurement error was tolerably small (see Appendix B in Supplemental materials for an example simulation). Moreover, because these discrepancies affected measurements at all ages, and because we were principally concerned with differences between age groups, they are unlikely to have affected the reported findings substantively.

Mean weight vectors were computed for each age group as the weighted arithmetic mean of each individual’s weight estimates. The arithmetic weighting was proportional to the number of trials the listener completed, because listeners who completed more trials would be expected to provide more well-constrained weight estimates. (Qualitatively similar, but noisier, results were found by simply excluding participants who fell below an arbitrary minimum number of trials cutoff.)

**Figure 5 fig5:**
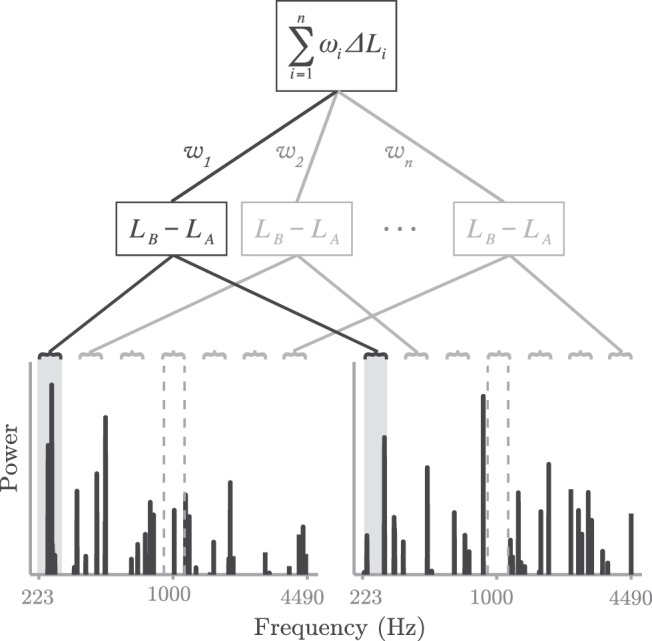
Graphical illustration of listeners’ assumed decision strategy (cf. Equation 1). The listener calculates the difference (in decibels) between equivalent spectral regions, and responds based on the linear-weighted sum of these values.

The mean weight vector for each age group was used to estimate each listener’s trial-by-trial decision variable (DV). To do this, it was assumed that listeners based their decision on the weighted sum of the difference in energy in each spectral bin, thus:

$$DV=\overset{n}{\underset{i=1}{\sum }}\omega_{i}▵L_{i}\,,$$ 1

where Δ*L* represents the difference in level (decibels) between the two stimulus observations, the subscript *i* denotes the spectral bin (of which there were seven, uniformly spaced along the stimulus range), and ω_*i*_ is the corresponding weight. An unbiased listener would respond “Interval 2” if DV was more than 0, and “Interval 1” otherwise. Such a model, shown graphically in Figure 5, was found previously to predict robustly the behavior of adults on this task (Lutfi et al., 2003; Tang & Richards, 2003), and it appeared to give a good account of listeners’ behavior in the present study.[Fig-anchor fig5]

Estimates of internal noise magnitude and bias were derived from psychometric fits to the probability of responding “Interval 2,” as a function of DV:

$$P(‘Interval2’)=\lambda_{\text{lo}}+(\lambda_{\text{u}\text{p}}-\lambda_{\text{lo}})\Phi (DV;\mu ,\sigma ),$$ 2

where λ_lo_ and λ_up_ are lower and upper asymptotes, and Φ(DV; μ, σ) is a cumulative Gaussian distribution with mean μ and standard deviation σ, evaluated at the values DV. Note that fitting psychometric functions to data gathered using an adaptive tracking procedure is not ideal, but has been shown to be valid for datasets similar to those reported here (Leek, Hanna, & Marshall, 1992). Fits were made using the PSIGNIFIT Matlab toolbox (Version 2.5.6), which implements the maximum likelihood method described by Wichmann and Hill (2001). The fitted value of the slope parameter, σ, was taken as a measure of internal noise magnitude, under the assumption that internal noise is additive and normally distributed. Note that σ is not sensitive to levels of multiplicative noise, substantial amounts of which would manifest as increased lapse rates and a skewed psychometric function that deviates from normality (Tyler & Chen, 2000). In keeping with the wider literature, substantial amounts of multiplicative noise were not apparent in the present data, and its role in development is assumed to be negligible.

Response bias was indexed by constant error—the point of subjective equality minus the point of physical equality on the psychometric function. As with weight efficiency, these measures yielded one estimate of bias and internal noise magnitude, per listener.

### Results and Discussion

**Figure 6 fig6:**
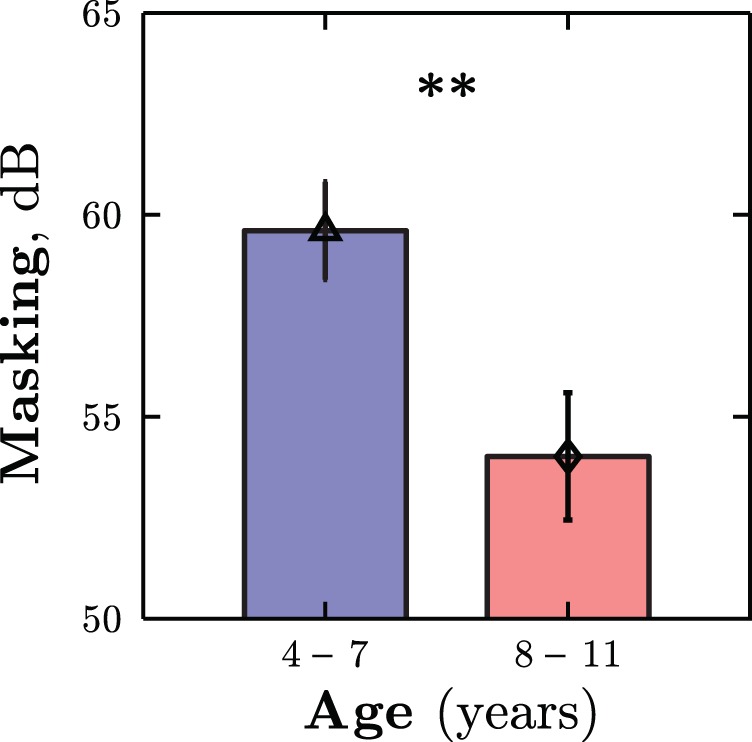
Experiment 2: Mean (± 1 *SE*) masking thresholds for younger and older children. See the online article for the color version of this figure.

The pattern of performance followed that of Experiment 1. No significant differences in DLs were observed between younger and older children in quiet, *t*(46) = 0.89, *p* = .379, but younger children exhibited significantly greater masking in the signal plus noise condition, *t*(46) = 2.86, *p* = .006 (see Figure 6). Note that the level of masking was greater for both age groups than in Experiment 1 (see Figure 6). This is likely to have been caused by the difference in stimuli: the maskers in Experiment 2 were spread over a much narrower spectral range (e.g., they were more likely to be similar in frequency to the target), and, by logarithmically distributing the noise, a potential pitch cue was removed (see the Experiment 2 Method section). [Fig-anchor fig6]

**Figure 7 fig7:**
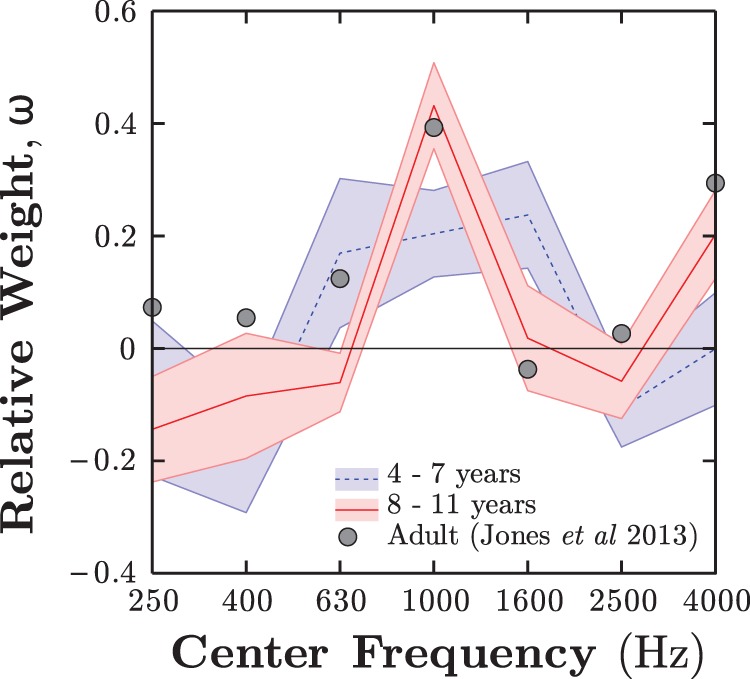
Experiment 2: Mean weight vectors (± 1 *SE*) for younger (blue, dashed) and older (red, solid) children. Circles give the mean, normalized weight coefficients from the untrained adults in Jones, Moore, Shub, and Amitay (2014), reproduced here for comparison. See the online article for the color version of this figure.

To explore why younger children were more adversely affected by noise, spectral weights were calculated for each individual. The group means (±1 *SE*) of these weight functions are shown for both ages in Figure 7. Younger children exhibited a flatter profile, giving greater relative weight to the spectral regions flanking the target (1 kHz). Conversely, older children produced a mean weight function that was closer to the ideal, and in good agreement with adult data presented by Jones, Moore, et al. (2014)—the results of which are reproduced in Figure 7 for comparison. Consistent with these group means, the average efficiency of the individual weight profiles was significantly greater in the older children than the younger, *t*(46) = 3.9, *p* < .001. Thus, older children more efficiently weighted the stimulus information, whereas younger children inappropriately attended to uninformative regions of noise; in particular, those that were similar in frequency to the target. [Fig-anchor fig7]

**Figure 8 fig8:**
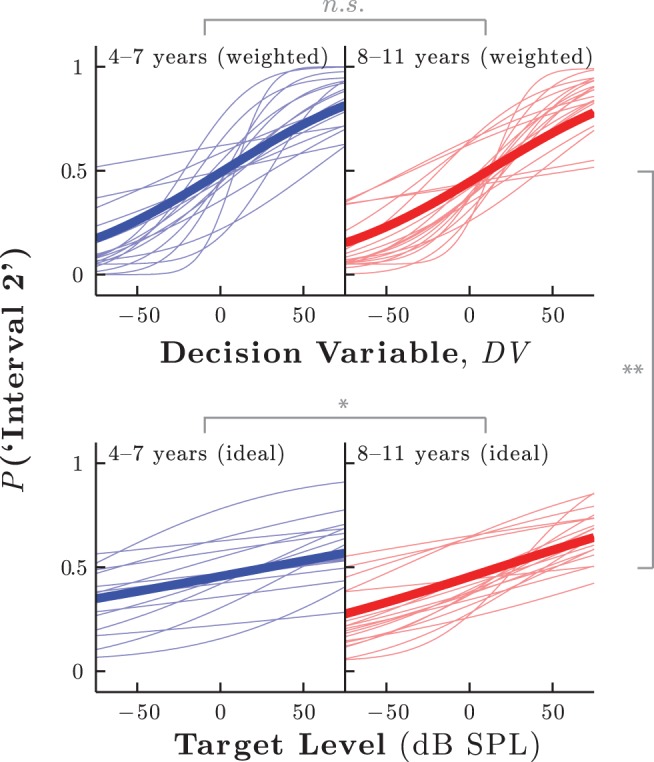
Experiment 2: Psychometric functions for younger (left) and older (right) children, fitted to Equation 2. The decision variable was the linear weighted sum of the trial-by-trial spectral energy, as per Equation 1. In Figure 8a, the weights are the empirical group mean values given in Figure 7. In Figure 8a, fits were made to the target level alone, which is equivalent to assuming an ideal weight vector (see Figure 2). See the online article for the color version of this figure.

Each listener’s trial-by-trial decision variable was computed with Equation 1, using the relevant group mean decision weights, ω (see Figure 7). To these values, each listener’s psychometric function was fitted (see Figure 8a), and estimates of internal noise magnitude and bias were derived. No significant differences in internal noise magnitude, *t*(32) = 0.91, *p* = .370, or bias, *t*(32) = 0.93, *p* = .363, were observed between age groups. [Fig-anchor fig8]

Alternate fits were also made without regard to the empirical weight data, by simply fitting responses to raw target level (see Figure 8, bottom panels). The results of these unweighted fits differed in two key respects from the weighted data described above. First, psychometric slopes were significantly shallower in both younger and older children (both *p* < .001), making levels of internal noise appear greater at all ages (see Figure 8a vs. Figure 8b). Second, younger children exhibited shallower slopes than older children, *t*(32) = 2.69, *p* = .011, suggesting relatively greater internal noise in younger children (see Figure 8b left vs. Figure 8b right). In short, these results indicate that if differences in attention (decision weights) are not accounted for, then changes in masking appear to be explained by differences in internal noise (psychometric slope). However, this “internal noise” account is poorer in a number of respects. Differences in internal noise cannot explain the different patterns of weights observed among younger children, and make no predictions as to how performance will change as the spectral distribution of the stimulus varies (see Experiment 3). Moreover, the unweighted fits tended to be more variable than the weighted fits. Thus, in the unweighted fits, there was substantially greater within-group variability in the slopes (*p* = .028) and points of subjective equality (*p* < .001) of individual listeners,^2^ and there was also a nonsignificant trend toward greater deviance between raw data and the fitted models, *t*(47) = −1.84, *p* = .081. Parsimony therefore favors the weighted model, in which internal noise played no substantive developmental role.

To sum up, Experiment 2 indicated that younger listeners were primarily limited by their ability to attend selectively to spectrally distributed information. In particular, 4- to 7-year-olds appeared less able to filter out information similar in frequency to the target sound, exhibiting elevated weights in the regions flanking the target. This is consistent with the notion of an “attention band” that narrows during development (Green, 1958; Lutfi, 1993). Unlike previous related work (e.g., Lutfi et al., 2003; Oh et al., 2001), other potential explanations for children’s poor performance—namely, changes in internal noise or response bias—could be discounted. Furthermore, by applying the method of reverse correlation, the shape of children’s attentional filter (i.e., how children distribute their attention across the audible spectrum) could be estimated with considerably more accuracy than previous methods have permitted (see Lutfi, 1993, for specific discussion on this point). Thus, it appeared that young children differed particularly in their inability to ignore information approximately one octave either side of the target frequency (see Figure 7).

## Experiment 3: Effects of Notch Widening

The attention band theory predicts that masking will be greater when the external noise is more similar in frequency to the signal. Previous data were grossly consistent with this prediction. Thus, masking was substantially greater in Experiment 2 than in Experiment 1, which used a wider range of maskers (≈ 5.6 octaves vs. ≈ 4.5 octaves). Conversely, masking was substantially less in Experiment 2 than was observed by Leibold and Neff (2007), who used a narrower range of (*N* = 10) maskers (≈ 3.3) and listeners of a similar mean age.

A more direct way to manipulate the similarity of signal and noise is to vary the size of the protected region around the target. As the protected region grows, the noise components will be forced farther from the signal. If the weight vectors in Experiment 2 are correct, then such an expansion should provide a particularly large release from masking in younger children, because they gave more weight to noise components proximal to the target (see Figure 7). Experiment 3 tested this by measuring masking levels in younger and older children, using four protected region widths. It was predicted that with narrow protected regions younger children would be disadvantaged relative to older children (replication of Experiment 2), but that this disadvantage would diminish as the width of the protected region increased (i.e., as the noise was progressively limited to regions that younger and older children weight equally).

A potential confound of this notch-widening approach is that a wider protected region will result in less masker variability (i.e., in terms of the trial-by-trial deviation in energy within each of the remaining spectral regions). With unpredictable noise, masker variability has been found to affect amounts of masking (Lutfi, 1993; Oh & Lutfi, 1998). Moreover, the changes in masking appear to interact with age, such that in low-variability conditions even very young children perform indistinguishably from adults (Oh et al., 2001). Thus, increasing the protected region is liable to abolish the developmental differences observed in Experiment 2 via a reduction in variability, independent of any differences in selective attention. Accordingly, in this experiment, the number of maskers was covaried with protected region width, so to maintain an approximately constant standard deviation of energy (decibels) in each spectral bin. The total level of the masker remained constant across all conditions (60 + ε dB SPL).

As an additional control, masking was assessed using a relatively predictable, broadband masker. If younger children continued to exhibit greater masking under these conditions, then this may indicate the presence of peripheral sensory deficits (e.g., such as broader auditory filters), which could provide an alternative account of the data in Experiment 2. No such differences were predicted, however, because the peripheral auditory system is almost (Irwin, Stillman, & Schade, 1986) or fully (Pujol, Lavigne-Rebillard, & Uziel, 1991; Schneider, Morrongiello, & Trehub, 1990) mature by early childhood.

### Method

#### Stimuli and design

The stimuli were the same as those in Experiment 2, except for the size of the protected region and the number of distractors, both of which were systematically covaried between blocks. As discussed above, a BBN condition was also added. The noise in this condition was equivalent to the 3,500 condition in Experiment 1, and consisted of a band-passed Gaussian noise with a rectangular notch at 891–1120 Hz. As in all other conditions, the noise was presented for 370 ms (including 20-ms cos^2^ on/off ramps), at a level of 60 + ε dB SPL.

The blocked conditions are shown in Table 2. The independent variable of interest was the width of the protected region. However, as discussed in Experiment 2: Methods: Stimuli and Design, the number of masker components (*N*) was covaried so to maintain an approximately constant level of energetic variability in each one-third octave bin. For every listener, the first block was always performed in quiet. The order of the remaining blocks was randomized.[Table-anchor tbl2]

### Results and Discussion

**Figure 9 fig9:**
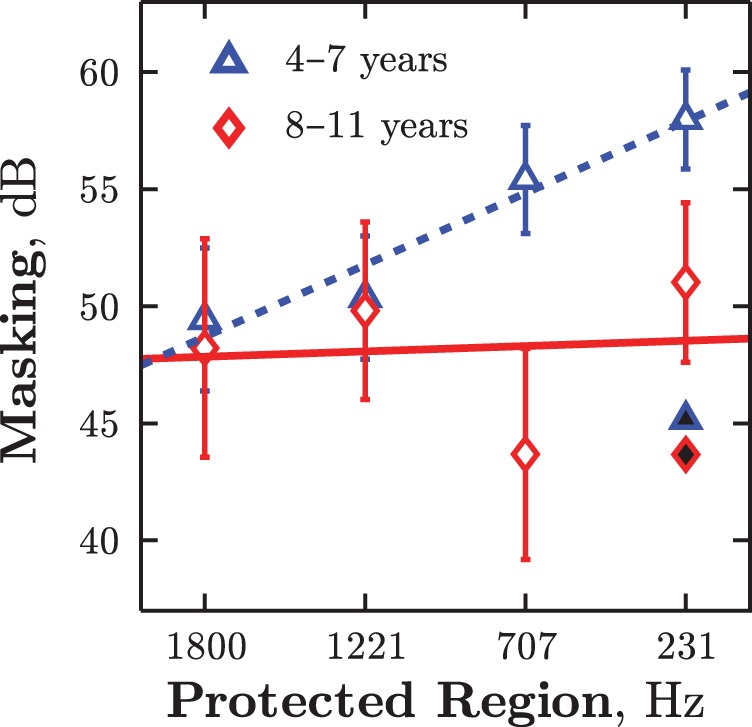
Experiment 3: Group mean masking levels (± 1 *SE*) as a function of protected region width, for younger and older children (see Table 2 for stimuli). Lines denote least mean square regression fits. Filled symbols give group mean masking in the notched broadband noise condition. See the online article for the color version of this figure.

As in Experiments 1 and 2, no differences were observed in quiet, *t*(52) = 0.41, *p* = .683, but younger children exhibited significantly greater masking in the noise conditions, *F*(1, 3) = 6.63, *p* = .011, η_p_^2^ = 0.03 (see Figure 9). Furthermore, masking decreased in younger listeners as notch width increased, *F*(3, 124) = 3.09, *p* = .030, η_p_^2^ = 0.01, but remained largely invariant in older children, *F*(3, 52) = 0.63, *p* = .597, as shown by the regression slopes in Figure 9. The Notch Width (within-subjects) × Age (between-subjects) interaction was significant in an unbalanced, mixed-effects ANOVA, *F*(3, 153) = 2.82, *p* = .041, η_p_^2^ = 0.02. Furthermore, as predicted by the weights in Experiment 2 (cf. Figure 7), the younger listeners were significantly poorer only in the two narrower notch conditions (both *p* ≤ .042), and did not differ from the older children in the two wider notch conditions (both *p* ≥ .610). [Fig-anchor fig9]

These data are consistent with the patterns of weights derived in Experiment 2. When the protected region was as narrow as in Experiment 2, younger listeners again exhibited greater masking. In contrast, when the protected region was increased (i.e., so to envelop those spectral regions that younger listeners weighted inappropriately), no differences were observed between older and younger listeners. This supports the notion that younger listeners are primarily impaired by their ability to ignore spectrally similar noise.

In BBN no difference in DLs was observed between age groups, *t*(45) = 0.27, *p* = .789. This indicates that auditory filters are not wider in younger children, and that differences in masking caused by unpredictable noise cannot be explained by peripheral mechanisms. It also suggests that the developmental differences in broadband masking observed in Experiment 1 were, as suspected, a consequence of the testing procedure.

#### Combined analysis: individual differences

In the presence of unpredictable noise, large individual differences in masking have often been observed (e.g., Lutfi et al., 2003; Neff & Dethlefs, 1995). The present study was no exception. For example, in Experiment 1, masking in the *N* = 30 condition varied by 40 dB in adults, and by 34, 33, and 58 dB in the three groups of children (ascending age), respectively. Previous authors have suggested that these individual differences may be larger in children than in adults (Lutfi et al., 2003; Wightman et al., 2003). However, we found no evidence of that. Thus, no heterogeneity of variance was detected between age groups in any of the six masking conditions in Experiment 1 (Levene’s test, *p*s > .05).^3^

To formally explore which factors predict individual differences in masking, multiple linear regressions were performed using the combined data from Experiments 2 and 3 (using the one common condition: *N* = 30; notch width = 231 Hz). No data from Experiment 1 were used, because, as discussed previously, significantly less masking was observed there than in the subsequent two experiments, *t*(36) = −8.55, *p* < .001. In addition, 25 of the remaining 102 children were excluded from all analyses because they did not complete the full battery of ancillary measures described in the General Method section (generally due to time constraints). The number of participants in all analyses was therefore 77.

**Figure 10 fig10:**
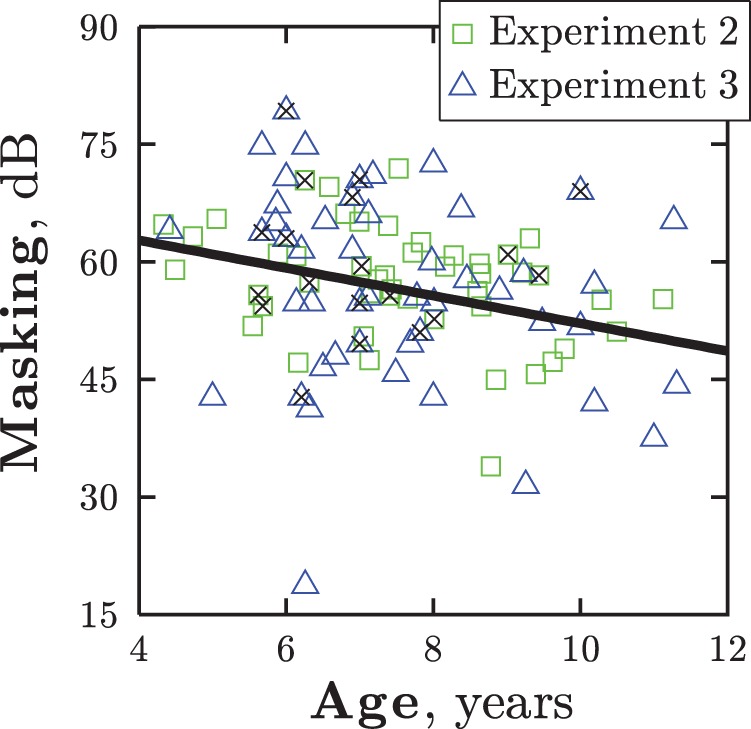
Experiments 2 and 3: Masking for individual observers as a function of age. The black line gives the least squares linear regression fit to the data. The data points marked with crosses were excluded from all analyses because data for one or more of the predictor variables (cf. Table 3, column 1) was missing. The point at 〈6.3, 18.8〉 was also excluded as a probable outlier. See the online article for the color version of this figure.

As expected, age was a significant predictor of masking, *F*(75) = 7.02, *p* < .001, *R*^2^ = 0.09, with masking decreasing by 1.8 dB per year (Figure 10). There was no benefit of adding sex to the model, indicating that amounts of masking did not differ between males and females. This is consistent with Oxenham, Fligor, Mason, and Kidd (2003), though contrary to Neff, Kessler, and Dethlefs (1996), who observed less masking in adult males than in adult females.[Fig-anchor fig10]

Levels of everyday attention, as assessed by the SWAN Scale, also failed to predict masking. This suggests there may not be a strong relationship between the level of general attentiveness that a child exhibits and his or her ability to selectively attend to task-specific information. It should be noted, however, that the SWAN was designed to identify children with abnormal attention, not to measure fine-grained differences in attention among the typical population; and only six individuals scored above the threshold of clinical concern. Even in these six individuals, however, there was no clear relationship with masking, with four lying in the 60th–82nd percentile, and two exhibiting *less* than average masking (23rd and 38th percentile).

Children living in more economically deprived neighborhoods had significantly greater amounts of masking (i.e., poorer performance in noise) than those of greater SES (*p* = .008). An association between SES and performance on such a basic sensory task is remarkable, but not unprecedented (e.g., Politzer, 1971). It is not likely to have been due to peripheral sensory deficits, because there was no association between SES and thresholds in quiet, *t*(75) = 0.93, *p* = .354, or in BBN (predictable), *t*(32) = 0.65, *p* = .521. At this point, we can therefore only speculate that the relationship between SES and masking may relate to differences in higher order factors, such as memory or concentration. Interestingly, no such relationship between SES and performance was observed in 1,469 children tested by D. R. Moore, Ferguson, Edmondson-Jones, Ratib, and Riley (2010), using a battery of simple auditory tests. This battery included various listening-in-noise tasks, but did not include any “unpredictable noise” tasks of the type we used in the present study. This difference may reflect the stronger cognitive demand of the present task, and is consistent with a general literature suggesting that high load on processes of cognitive control can lead to increased distractor interference (Lavie, Hirst, de Fockert, & Viding, 2004).

Finally, in the present study, there was also a nonsignificant relationship between vocabulary (BPVS) and masking, which became significant when SES was included in the model (*p* = .037).

Thus, the best full model included age, SES, and vocabulary as predictors (see Table 3), and explained 26% of the variance in masking between individuals, *F*(3, 72) = 8.56, *p* < .001, *R*^2^ = 0.26. This compares favorably with a recent large-scale study of auditory development, where 20% of variability in speech-in-noise identification was accounted for by a battery of 96 measures (D. R. Moore et al., 2010). However, it remains an interesting and open question as to what explains the majority (74%) of the variability in performance between individuals.[Table-anchor tbl3]

## General Discussion

This work aimed to show why children struggle to hear sounds in the presence of unpredictable noise. By applying adult psychophysical techniques to children, the relative contributions of internal noise and selective attention could be assessed within a unified framework, and the decision strategies of younger and older children could be quantified.

The sole mechanism of developmental change was shown to be selective attention. Older children (8–11 years old) were similar to adults in their ability to ignore irrelevant information. In contrast, younger children (4–7 years old) were less able to filter out (i.e., gave greater weight to) noise that was similar in frequency to the target tone, despite performing as well as adults in quiet. This result provides novel and direct evidence that younger children struggle to hear in unpredictable noise because of poor selective attention (e.g., as suggested by Leibold & Neff, 2007; Lutfi, 1993).

This conclusion is consistent with a general literature that has indicated attention improves substantively within the first 7 years of life (e.g., Lane & Pearson, 1982; Ruff & Rothbart, 1996), as well as with more specific auditory studies that have indicated frequency selectivity is diminished in infants (Bargones & Werner, 1994) and preschool children (Stellmack, Willihnganz, Wightman, & Lutfi, 1997), but is relatively mature in 7-year-old children (Greenberg et al., 1970). Notably, several of these latter studies used the target probe paradigm, which, as described in the Introduction section, measures the tendency to neglect unexpected stimuli. In this light, the deficits of the 4- to 7-year-old children reported in the present study may actually represent an adaptive strategy: a developmental principle to exclude as little sensory information as possible. Such a strategy is suboptimal for the present detection task, but in other situations will reduce the likelihood of missing unexpected information. For example, a wide attention band would be particularly useful for bootstrapping development in situations where the important information is not self-evident (see Gureckis & Love, 2009).

An unanticipated finding of note was that children from lower socioeconomic backgrounds exhibited greater masking in unpredictable noise conditions (but not in quiet or in predictable noise). This could not be explained by peripheral sensory deficits, and is consistent with recent reports that children from lower socioeconomic backgrounds exhibit “a reduced ability to filter irrelevant information” (see Stevens, Lauinger, & Neville, 2009, p. 634), and generally perform poorly on tests of selective attention (Lupien, King, Meaney, & McEwen, 2001; Mezzacappa, 2004) and executive control (Noble, McCandliss, & Farah, 2007).

### Implications

With respect to our understanding of hearing in noise, the present work suggests that young children’s difficulties hearing-in-noise can be obviated if the similarity between signal and noise is reduced by approximately one octave or more. This is particularly encouraging, because previous studies (Hall et al., 2005; Wightman et al., 2003) have indicated that children not only exhibit greater masking, but that, compared with adults, they are relatively poor at exploiting “attention cues” such as target-masker spatial differences to reduce this masking.^4^ The reduction in masking observed in Experiments 1 and 2 is consistent with these results, because it suggests that, although the children in Experiment 1 were able to make *some* use of the inadvertent pitch cue contained therein, this effect was only partial, and a substantial deficit remained (relative to older children and adults). Crucially though, the results of Experiment 2 predicted (and Experiment 3 confirmed) that the difficulties of younger children are confined specifically to those noises that are: (a) unpredictable (i.e., like a human voice and unlike a running motor), and (b) similar in frequency (i.e., pitch) to the target. Thus, although the deficits in selective attention seen in younger children may be inevitable, it appears that their deleterious consequences could be avoided, either by removing noises similar in content to the target, or even by using a steady BBN to mask more variable noises. This may be of particular interest to researchers in educational psychology, where deficits in selective attention are of particular concern within classrooms, and have been linked to academic performance across a range of tasks (see Stevens et al., 2009, for an overview).

The present work also has implications for how we understand the role of internal noise (random variability due to, e.g., stochastic neural activity) during auditory development. First, it suggests that internal noise does not limit children’s ability to hear in noise. More generally though, it brings into question the importance of internal noise as an explanatory construct and the validity of its measures. Thus, previous studies (e.g., Buss et al., 2006; Jones et al., 2013) have taken psychometric slope as an index of internal noise magnitude. Experiment 2 demonstrated that this is not always valid, because apparent differences in psychometric slope were fully explained by differences in the decision strategies (i.e., spectral weights) of younger and older children.^5^ Furthermore, the decision strategy account was superior to the internal noise account, having both greater explanatory and predictive power. This shows that developmental changes in internal noise cannot be directly inferred from changes in psychometric slope, as was done by Buss et al. (2006), and that potential differences in decision strategy must also be accounted for. This does not mean that the claim of some authors—that internal noise drives the maturation of performance on some auditory tasks—is necessarily incorrect. For example, it may be that, as previously argued (Watson, Kelly, & Wroton, 1976), tasks with minimal stimulus uncertainty somehow “permit” selective attention, in a way that the present task does not. In this case, internal noise may indeed determine performance on more basic auditory tasks, whereas on more complex tasks the listener’s decision strategy becomes increasingly the limiting factor. Alternatively, it may be that, even with simple auditory tasks, the poorer performance of young children could be explained by differences in decision strategy, in which case internal noise has been overestimated in the wider developmental literature, both in magnitude and importance. To test these hypotheses would require decision strategies to be explicitly quantified on a range of auditory judgment tasks, for example, by applying analogs of the trial-by-trial reverse correlation method used in the present study. However, what is clear already is that, in at least in some circumstances, internal noise is redundant when explaining sensory development, and that it should be avoided where possible in favor of accounts with greater explanatory power.

Finally, it may be instructive to contrast the present result—that young children gave excessive weight to uninformative information channels—with recent suggestions that sensory integration is largely absent in young children. For example, Gori, Del Viva, Sandini, and Burr (2008) observed that children prior to 8 years old fixated on a single cue on combined visual/haptic discrimination tasks (see also Nardini, Bedford, & Mareschal, 2010, for an analogous result within a single modality). Gori et al. (2008) argued that their results reflected a developmental imperative not to integrate sensory information, in order to provide independent estimates for cross-calibration. Although we cannot rule this out for multisensory perception, the present data are inconsistent with this account as a general principle, because younger children actually integrated over a broader range of information channels (as previously shown in infants by Bargones & Werner, 1994). It may therefore be that both these phenomena—sensory dominance and the failure to attend selectively shown here—reflect a more general inability to appropriately weight competing sources of information. Children may simply find it more difficult to learn which aspects of the stimulus are relevant (or to direct attention toward the relevant components once they are apparent), and may thus underweight task-relevant information, or overweight task-irrelevant information. Whether this reflects a diminution of capacity, or whether some children just require more practice to learn the structure of the task, remains an open question. However, it is interesting that adults can learn to improve how they weight sensory information with practice (Jones, Moore, et al., 2014), and that even some 6- to 8-year-old children can be trained to make auditory discrimination judgments as well as adults (Halliday, Taylor, Edmondson-Jones, & Moore, 2008). It is therefore tempting to speculate whether “sensory development,” over the age range of maturation reported here (4–8 years), may at times represent an improvement in learning, with better performers able to learn more quickly what the task-relevant information is, and/or how to attend to it.

### Limitations and Future Directions

This study relies heavily on methods (e.g., reverse correlation) developed originally using highly trained adult participants. The decision processes of such listeners are likely to be relatively consistent across trials, and a large number of trials can be gathered, further minimizing the effects of occasional aberrations in judgment. The extent to which such psychophysical can be applied to children with no prior experience was largely unknown prior to the study, and so due caution is advised. However, appropriate steps were taken where possible to safeguard against spurious results, including the use of simulations (see Appendix B in Supplemental materials), control experiments, and relatively large sample populations. Furthermore, the internal consistency of the present results (comparing across experiments) is particularly encouraging, and suggests that reverse correlation could be a highly productive tool with which to study developmental psychology, just as it has been in the adult psychophysical literature (see Dai & Micheyl, 2010, for an overview). However, we note that, on the basis of anecdotal pilot work, it was particularly important to keep the observer vigilant and engaged with the task. In this respect, the extensive use of child-friendly graphics and animations may have been particularly valuable for eliciting reliable measurements.

In terms of scope, it is important to note that the present study focused principally on mechanisms that may limit a child’s perceptual sensitivity. However, children’s performance on tests of sensory judgments may also be limited by a priori (nonsensory) inefficiencies, such as bias and inattentiveness (e.g., Viemeister & Schlauch, 1992; though cf. Schneider, Trehub, Morrongiello, & Thorpe, 1989). Regarding inattentiveness, we did not observe a relationship between masking and the SWAN Scale. However, the SWAN is a relative coarse measure, and we cannot rule out more nuanced effects of inattention. For example, D. R. Moore, Ferguson, Halliday, and Riley (2008) observed that some children may initially perform a psychophysical task well, but subsequently lose interest once the task becomes difficult. Such children are liable to exhibit V-shaped adaptive staircases, making mistakes later in the test block in response to stimuli that they formerly discriminated with ease. Similar behavior was observed anecdotally in the present study, and may have limited the performance of some children. However, there are, as yet, no established procedures for rigorously quantifying such nonstationary behavior, and doing so would be particularly challenging with the present data because of the need to account for the substantial trial-by-trial variations in the stimulus. Regarding bias, although no preference for either response alternative was apparent, children often verbalized *contingent* response preferences, such as “[the target] was on the left last time, so this time it will be on the right.” We have also observed such behavior in unpracticed adults, where it is liable to elevate thresholds in a manner akin to reduced sensitivity (Jones, Moore, & Amitay, 2014). It remains an interesting and open question to what extent such nonsensory factors limit children’s performance on tests of sensory judgment, and whether changes in bias and inattention can explain apparent maturation of sensory processes.

### Conclusion

1. Masking by unpredictable noise was shown to decrease during childhood, becoming adult-like by 9–11 years (Experiment 1).

2. Reductions in masking were explained by improvements in selective attention. Younger children overweighted bands of noise that were similar in frequency to the target signal (Experiment 2). Moreover, they performed like older children once these portions of the stimulus were removed (Experiment 3). In particular, younger children appear to be selectively poorer at ignoring distracting information that lay within approximately ± 1 octave of the target in frequency.

3. Other potential explanations of children’s poorer performance (i.e., internal noise, bias, or peripheral sensory deficits) were shown to play no role in children’s development. In particular, apparent changes in internal noise could be fully accounted for by differences in selective attention. Thus, once changes in selective attention were accounted for, no differences were observed between the psychometric functions of younger and older children (Experiment 2).

4. In addition to age, both SES and verbal ability were shown to account for a significant proportion of individual variability in masking.

## Supplementary Material

10.1037/a0038570.supp

## Figures and Tables

**Table 1 tbl1:** Participation Details for All Children

	Age, years		*N*		
Experiment	Range	*M*	Total	Excluded^a^	Male
1	5–6	6.1	18	7 (2)	6
	7–8	8.0	18	1 (0)	9
	9–11	10.1	13	0 (0)	6
	Total		49	8 (2)	24 (43%)
2	4–7	6.5	35	7 (7)	13
	8–11	9.1	24	4 (2)	11
	Total		59	11 (9)	24 (50%)
3	4–7	6.6	56	18 (10)	18
	8–11	9.7	23	7 (2)	9
	Total		79	25 (12)	27 (50%)
**Grand**	**Total**		**187**	**44 (23)**	**75 (47%)**
*Note.* For clarity, only the sex of included participants is given. Note that: (a) some of the children excluded because of incomplete data were nonetheless eligible for inclusion in the final combined analysis of individual differences; and (b) the higher noncompletion rate in Experiment 3 was due principally to a reduction in the amount of testing time available per child.					
^a^ Values in parentheses provide the number of listeners who were excluded post hoc, based on their hearing threshold in quiet.					

**Table 2 tbl2:** Experiment 3 Stimulus Conditions

	Protected region (Hz)		
*N* components	Lower	Upper	Width
0 (quiet)	NA		
15	445	2245	1800
25	561	1782	1221
25	707	1414	707
30	891	1122	231
715 (BBN)^a^	891	1122	231
*Note.* Tones were drawn from a log-uniform distribution within the range of 223−4490 Hz, excluding a protected region geometrically centered on the target (within which no masker energy was permitted to fall). BBN = broadband notched noise; NA = not applicable.			
^a^ Condition equivalent to a BBN.			

**Table 3 tbl3:** Improvements in a Linear Regression of Masking on Age, When a Second Variable Was Added to a Model

Independent variable	*t*_74_	*p*	β	Δ*R*^2a^
Sex	−0.37	.709	−0.76	0.00
SES	−2.73	.008	−10.65	0.08
BPVS	−1.99	.051	−0.10	0.04
SWAN	1.37	.175	1.55	0.02
*Note.* ADHD = attention-deficit/hyperactivity disorder; BPVS = British Picture Vocabulary Scale; SES = socioeconomic status; SWAN = Strengths and Weaknesses of ADHD symptoms and Normal behavior Scale.				
^a^ The increase in explained variance (*R*^2^) relative to a model containing age alone.				

## Footnotes

1 Note that although internal noise is often equated solely with neural noise, the behavioral models used to measure internal noise seldom make or enforce this assumption. Also note that sources of internal noise, such as blood flow, may actually be highly periodic and nonrandom. Nonetheless, their effects may be largely uncorrelated with the sensory judgment task, and so may be considered a source of random variability.

2 Variability was indexed by standard deviation. Paired bootstrapping (*N* = 2,000) was used to derived sampling distributions for mean standard deviations with and without weights. Equality was tested by computing the difference in *z* score, which was converted to a *p* value using the normal distribution (Altman & Bland, 2011).

3 Similarly, with the combined Experiment 2 and 3 data, there was no indication that within-age-group variability decreased with age. Thus, a rolling age window, 3 years in width, yielded standard deviations ranging from 8.4−9.5 dB of masking, with similar variability among the youngest (9.5 dB) and oldest (9.4 dB) listeners.

4 Though some authors have reported benefits of spatial separation on children’s masking (Litovsky, 2005), there is some evidence that temporal cues may be effective at guiding attention even in young children (Hall et al., 2005; Leibold & Neff, 2007).

5 The insight that changes in selective attention may affect the slope of the psychometric function is not a new one. For example, see Allen and Wightman (1994) for a discussion of this point.
