# Supplementary material for: Development of Auditory Selective Attention: Why Children Struggle to Hear in Noisy Environments
Source: Dev Psychol. 2015 Mar;51(3):353–69. doi: 10.1037/a0038570 (PMC4337492; doi:10.1037/a0038570)
Supplement: Supplementary file 1 [file DEV-DEV2-Jones20143167-RR-F22.pdf]

Appendix A
Detection thresholds in quiet

Absolute detection thresholds for all experiments are given in Table A1, broken down by age. Although there was a general trend for younger children to exhibit higher thresholds, in no cases were the differences between groups significant [all $p > 0.05$]. Furthermore, the group-differences in quiet thresholds were trivial in size compared to the differences in masking, which were typically an order of magnitude greater.

Also shown in Table A1 are the number of individuals excluded based on their threshold in quiet (N.B.: additional children were excluded due to missing data, caused other by a lack of time or non-compliance. These data are not shown here). Exactly why some individuals performed poorly is unknown. Acute hearing problems were evident in a minority of individuals, and mild hearing loss due to non-cognitive etiologies is not uncommon in children (Zielhuis, Rach, van den Bosch, & van den Broek, 1990). However, in many cases inattentiveness or a lack of interest provided a more parsimonious explanation. Exactly how such general inattentiveness relates to more specific deficits in selective attention (if indeed it does) is an interesting question, but one that falls outside the scope of the present study.

			Threshold (dB SPL)	
Experiment	Age	n	μ	σ
Experiment I	5 – 6	11 (2)	12.5	3.9
	7 – 8	17 (0)	10.4	5.7
	9 – 11	13 (0)	11.4	5.0
	adults	15 (0)	8.6	6.5
Experiment II	4 – 7	28 (7)	13.0	5.8
	8 – 11	20 (2)	11.6	5.0
Experiment III	4 – 7	38 (10)	9.9	5.2
	8 – 11	16 (2)	8.9	4.0

Table A1. Means and standard deviations for absolute detection thresholds in quiet, across all Experiments. Figures in parentheses indicates the number of additional children who were excluded from all analyses based on the detection thresholds in quiet (> 20 dB HL; see General Methods for details). These individuals were not included in the calculation of group mean threshold, μ, or its standard deviation, σ.

Appendix B
MATLAB code for weight estimation simulations

The listing below provides code in which decision weights are repeatedly estimated using multiple logistic regression, given a simulated observer with no response bias and a known weighting function. This allows estimates of decision weights to be assessed as a function of the paradigm (number of trials), stimulus (target level and standard deviation), and observer (weight function, internal noise magnitude). Note that the simulation is only a simplified approximation to the present experiment, and some aspects (e.g., the adaptive tracking algorithm) have been omitted for clarity.

```
function [] = exampleWeightsSim()
% simulate weight estimates, under various conditions

    % 1. User params
    clear all
    weights         = [0 0 .25 .5 .25 0 0]; % sum to 1
    wIdeal          = [0 0 0   1  0   0 0]; % ideal weights
    targLvl         = [30 15];             % target [mean std] (in dB)
    nTrials         = 500;                  % per weight measurement
    nSimulations    = 1000;                 % n Monte Carlo simulations

    % 2. Estimate weights
    wEst = nan(nSimulations, length(weights));
    efficiencyEst = nan(nSimulations, 1);
    efficiencyAct = nan(nSimulations, 1);
    for i=1:nSimulations
        % estimate weights
        wEst(i,:) = estimateWeights(targLvl, weights, nTrials);
        % compute rms efficiency
        efficiencyEst(i) = 1 - sqrt(mean((wIdeal - wEst(i,:)).^2));
        efficiencyAct(i) = 1 - sqrt(mean((wIdeal - weights).^2));
    end

    % 3. Plot data
    set(0,'DefaultTextInterpreter','latex'); % init plot options
    figure('Units','centimeters', 'Position',[10 10 25 6]);
    hold on;
        stem(weights);
        plot(wEst(i,:), 'k:');
        text(1,1, sprintf('$\\eta_{Est}$ = %1.2f', efficiencyEst(i)));
        text(1,.8, sprintf('$\\eta_{Act}$ = %1.2f', efficiencyAct(i)));
    hold off;
    xlim([0 length(weights)+1]); ylim([-.3 , 1.3]);
    xlabel('N'); ylabel('Weight, $\omega$');
end

function [wEst, pc] = estimateWeights(targLvl, weights, nTrials)
% estimate weights using multiple logistic regression

    % init
    nosieLvl = 65; % corresponds to ~6 dB Std Dev per channel
    intNoise = 2; % arbitrary units
    targIdx = floor(length(weights)/2)+1;

    % generate notched noise
    x = rand(nTrials,length(weights),2);
    x(:,targIdx,:) = 0;
    x = bsxfun(@times, x, nosieLvl/sum(x,2));
    % add target
    targInt = (rand(nTrials,1)>.5)+1;
    targ = max(0, targLvl(1)+randn(nTrials,1)*targLvl(2));
    x(targInt==1,targIdx,1) = targ(targInt==1);
    x(targInt==2,targIdx,2) = targ(targInt==2);
    % compute level difference
    Int1 = x(:,:,1);
    Int2 = x(:,:,2);
    lvlDiff =  log10(max(Int2,1)) - log10(max(Int1,1));

    % add internal noise
    noiseSamples = bsxfun(@times, randn(size(lvlDiff)), intNoise);
    internalDiff = lvlDiff + noiseSamples;

    % calculate resposnes
    c = sum(bsxfun(@times, internalDiff, weights), 2);
    respInt = (c > 0) + 1; % unbiased criterion
    anscorrect = targInt==respInt;
    pc = mean(anscorrect); % percent correct

    % fit weights
    b = glmfit(lvlDiff,respInt-1,'binomial','link','logit');
    wEst = b(2:end);                    % remove constant term
    wEst = wEst .* 1/sum(abs(wEst));    % normalise to sum to one
end
```

Listing 1: Code for fitting decision weights to simulated data using Reverse Correlation
